# Resveratrol Inhibition of Cellular Respiration: New Paradigm for an Old Mechanism

**DOI:** 10.3390/ijms17030368

**Published:** 2016-03-17

**Authors:** Luis Alberto Madrigal-Perez, Minerva Ramos-Gomez

**Affiliations:** 1 Laboratorio de Biotecnología Microbiana, Ingeniería Bioquímica, Instituto Tecnológico Superior de Ciudad Hidalgo, Ciudad Hidalgo 61100, Mexico; lmadrigal@itsch.edu.mx; 2 PROPAC, Facultad de Química, Universidad Autónoma de Querétaro, Cerro de las Campanas S/N, Queretaro 76010, Mexico

**Keywords:** resveratrol, cellular respiration, molecular mechanism, energy homeostasis, antioxidant, mitochondrial dysfunction

## Abstract

Resveratrol (3,4′,5-trihydroxy-*trans*-stilbene, RSV) has emerged as an important molecule in the biomedical area. This is due to its antioxidant and health benefits exerted in mammals. Nonetheless, early studies have also demonstrated its toxic properties toward plant-pathogenic fungi of this phytochemical. Both effects appear to be opposed and caused by different molecular mechanisms. However, the inhibition of cellular respiration is a hypothesis that might explain both toxic and beneficial properties of resveratrol, since this phytochemical: (1) decreases the production of energy of plant-pathogenic organisms, which prevents their proliferation; (2) increases adenosine monophosphate/adenosine diphosphate (AMP/ADP) ratio that can lead to AMP protein kinase (AMPK) activation, which is related to its health effects, and (3) increases the reactive oxygen species generation by the inhibition of electron transport. This pro-oxidant effect induces expression of antioxidant enzymes as a mechanism to counteract oxidative stress. In this review, evidence is discussed that supports the hypothesis that cellular respiration is the main target of resveratrol.

## 1. Introduction

The polyphenol resveratrol (3,4′,5-trihydroxy-*trans*-stilbene, RSV) is a phytochemical found in over 70 species of plants, including plants of economic importance such as grape (*Vitis vinifera*), cranberry (*Vaccinium macrocarpon*), and peanut (*Arachis hypogaea*). Exposure of these plants to biotic and abiotic stresses induces RSV synthesis. Nonetheless, RSV production is mainly associated with plant-pathogenic attacks [1,2,3]. In this regard, RSV biological significance in plants has been linked to the decrease of fungal cell viability to counteract pathogenic cellular proliferation [4,5]. However, the exact mechanism by which RSV disturbs cell viability is still unclear. The inhibition of the electron transport chain (ETC) and the F_0_F_1_-ATPase is one of the promising hypothetical-mechanisms of RSV action [6,7]. This idea could explain the proliferation inhibition of undesirable pathogenic organisms by lessening its cellular energy production and the health benefits exerted in mammalian systems including its antioxidant properties. However, evidence supporting this concept is still insufficient. The mechanism underlying toxic and beneficial effects of RSV has not been fully elucidated. Therefore, some authors propose that RSV could activate diverse proteins involved in different signaling pathways [8,9] indicating the diverse nature of this molecule. On the other hand, other studies reveal that adenosine monophosphate (AMP) protein kinase (AMPK) is the main target of RSV, supporting the hypothesis of a single signaling pathway with a pleiotropic effect [6,10,11]. Nonetheless, how RSV activates AMPK remains unclear and this could be the key to understanding RSV mechanism. It is reasonable that increase of AMP levels by RSV-mediated inhibition of cellular respiration promotes AMPK activation, although this idea was not fully confirmed [6]. In this review, evidence is discussed that supports the putative AMPK signaling pathway activated by RSV due to the inhibition of cellular respiration and its relationship with amelioration of some molecular processes related to metabolic disorders including chronic-degenerative diseases (CDD).

## 2. Biological Significance of Resveratrol in Plants

In order to establish the molecular mechanism of the well-known beneficial effects of RSV in mammalian systems, it is important to note that the biological significance of RSV in plants is related to an environmental defense mechanism. This is key phenotype can help researchers to better understand RSV properties [12]. Several studies have demonstrated that RSV is produced in response to biotic and abiotic challenges [3,13]. However, the toxic effect on plant-pathogenic fungal organisms is the outstanding purpose of RSV in plants [14,15,16]. Therefore, when hyphae penetrate through the epidermis of the plant, it promotes the production of proteins and carbohydrates that elicit a plant response. Thus, the plant reacts blocking or delaying the advancement of the invader microorganism producing antifungal compounds such as RSV, which acts as a phytoalexin [2,17,18]. Similarly, cell-wall hydrolysates of pathogens were effective to elicit a similar response [3]. The rapid accumulation of these phytoalexins in the vicinity of the pathogen attack is critical for plant defense [19]. Furthermore, there is a strong correlation between the tolerance of fungal strains to phytoalexin and their pathogenicity on the plant host [12,19]. For instance, the localization and concentration reached by RSV is critical to inhibit fungal growth [20]. In this regard, it has been demonstrated that the enzyme stilbene synthase, which is a limiting-step enzyme in RSV biosynthesis, was predominantly expressed in the exocarp of grape berries, which correspond to the highest levels of RSV found in this particular tissue of the berry [20]. The effective RSV concentration to inhibit the growth of *Botrytis cinerea* ranges from 60 to 160 µg/mL [21], which corresponds to RSV levels in grape skins ranging from 19 to 508 µg/g [22]. Others have reported an average RSV content of 65.67 µg/g [23]. Altogether, this data indicates that RSV synthesis is induced in plant tissues under pathogenic attack, which provides a defense mechanism to counteract pathogen proliferation in the plant.

## 3. Molecular Mechanism of Resveratrol Toxicity

Although the RSV synthesis is elicited to counteract fungal infection, the molecular mechanism of the RSV anti-fungal action is still unclear. The lipophilic properties of RSV suggest that the site of RSV action could reside within the membranes. It has been demonstrated that RSV penetrates the membranes and localizes them in the hydrophobic acyl region near the polar headgroup [24,25]. Furthermore, *B. cinerea* conidia treated with RSV demonstrated a disruption in the plasma membrane and the mitochondria exhibited a complete disorganization of the cristae [21]. This implies a strong correlation between RSV membrane localization and shape defects in these cells. Indeed, the addition of 60 to 100 µM RSV caused several changes in morphology of erythrocytes and human embryonic kidney cells [24,26]. Hence, these results suggest a highly conserved effect of RSV at least in Eukaryotic organisms.

**Figure 1:**
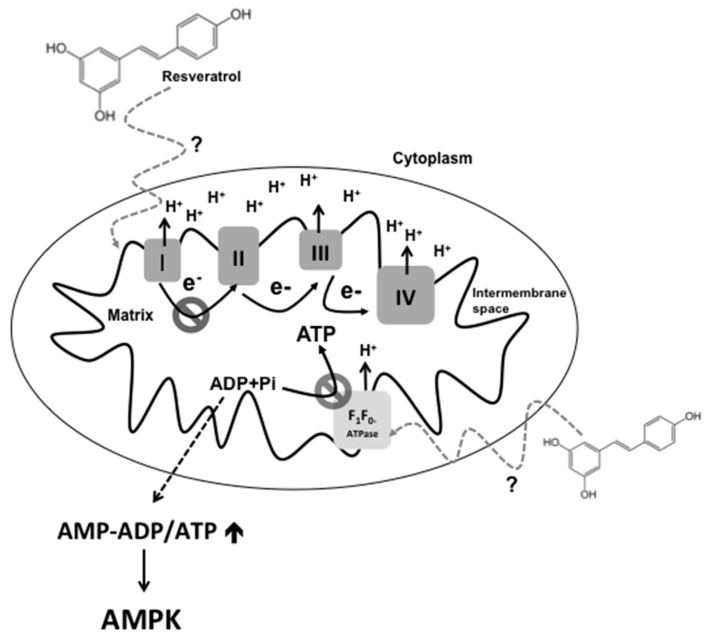
RSV inhibits mitochondrial respiration by a possible interaction with mitochondrial membranes and/or FoF1-ATPase. The possible interaction between RSV and the mitochondrial membrane would disrupt the association of the complex I (I) within the membrane, which, in turn, could inhibit the activity of complex I. On the other hand, RSV inhibits F_1_F_0_-ATPase activity; this might decrease the amount of ATP generated and increase the levels of AMP, which allow the activation of AMPK. Complex II (II), complex III (III) and complex IV (IV). The dashed line and question mark denote an unresolved molecular mechanism.

Furthermore, RSV also inhibits the activity of protein kinase C α (PKC α) in liposomes for which, localization and activity is associated with membranes, indicating that RSV disturbs the interaction of the membrane with the this protein [27,28]. In this regard, other membrane-associated proteins such as F_0_F_1_-ATPase and ETC proteins are also inhibited by RSV [7,29]. For example, RSV lessens about 20% of complex III activity [30]. Furthermore, RSV also inhibits F_0_F_1_-ATPase activity in rat brains and livers (IC_50_ of 12–28 µM) [30,31]. Interestingly, RSV did not exert a significant effect on the NA^+^/K^+^-ATPase activity of porcine cerebral cortex [31]. This indicates a specific action of RSV on the mitochondrial membrane or F_0_F_1_-ATPase protein. Therefore, it has been proposed that RSV disturbs the energy metabolism of the infectious pathogen. Thus, the molecular mechanism of RSV toxicity has been related to the suppression of cellular respiration due to membrane damage. The first studies of the mechanism of resveratrol actions demonstrated inhibition of cellular respiration in cell lines by this stilbene, and this phenotype has also been described in *Saccharomyces cerevisiae* [32] and in mammalian systems [33]. In addition, F_0_F_1_-ATPase inhibition by RSV has also been demonstrated in *Escherichia coli* [34]. Therefore, these reports suggest a highly conserved mechanism of action of RSV on cells, which might impact energy metabolism. This could explain the toxic effect of RSV. It has been proposed that RSV might elicit the same effect in eukaryotic cells, due to the highly conserved network of central metabolism in these organisms. The disruption by RSV of the principal energy production pathway perturbs cellular ATP homeostasis in eukaryotic cells. It has been reported that RSV decreases the respiratory control ratio in rat brain mitochondria (EC_50_ of 24.5 µM) [30]. This indicates that RSV might disturb the ATP production associated with ETC. As expected, RSV supplementation increases AMP/ADP levels relative to ATP, as reported in C_2_C_12_ myotubes [35] and Hep-G2 cells [36], both treated with 50 µM RSV. Presumably, this high AMP-ADP/ATP ratio induced by RSV could stimulate the activation of the AMPK protein [36] (Figure 1**)**, which is the well-known target of RSV in mammals. Therefore, the activation of AMPK could explain the pleiotropic effect of RSV and its health benefits.

## 4. Relation between AMPK and Resveratrol

The activation of AMPK by RSV might promote profound changes in various cellular processes including mitochondrial biogenesis, autophagy, lipolysis, and stress responses, among others [37]. Several studies have established the essential role of AMPK in the effective regulation of energy metabolism, which is a crucial requirement for cellular homeostasis [38]. In mammalian systems, after activation, AMPK stimulates energy production from glucose and fatty acids during stress and inhibits energy consumption for protein, cholesterol, and glycogen synthesis [37,38]. Those effects have also been reported in RSV supplementation in mammalian systems. The dietary restriction (DR) stimulates AMPK activity similar to RSV, while nutritional overload seems to impair it and simultaneously induce insulin resistance in many tissues. Thus promoting the appearance of the components of the metabolic syndrome, obesity, diabetes, and cardiovascular diseases [39]. Currently, AMPK is considered an important drug target and its novel activators may be useful in the therapy of metabolic and neurodegenerative diseases [40]. Therefore, it is expected that activation of AMPK by RSV promotes all physiological and molecular changes related with the health properties attributed to the AMPK function.

The activation of AMPK by RSV has been demonstrated by *in vitro* and *in vivo* studies, and it has been reported that RSV can ameliorate several CDDs via this mechanism. For example, RSV supplementation at 400 mg/kg/day increases insulin sensitivity and reduces fat accumulation by up to 40% in rats fed with a high-fat diet [41]. Moreover, these effects were nullified in rats with the *AMPK^α1−^* and *AMPK^α2−^* phenotype, indicating that AMPK mediates the effect of RSV [11]. Nevertheless, the mechanism by which RSV activates AMPK is not clear, and it has also been proposed that Sirt1 could be the main target of RSV [42,43]. This hypothesis has gained importance since Sirt1 activation allows both the deacetylation and activation of liver kinase B1 (LKB1) [44], which, in turn, phosphorylates and activates AMPK [45]. Nevertheless, other studies demonstrate that RSV-mediated AMPK activation could be the result of competitive inhibition of cAMP phosphodiesterases (PDE4), leading to increased intracellular concentration of cAMP and activation of AMPK via the cAMP-regulated guanine nucleotide exchange factor (EPAC1) [8] in a Sirt1-independent manner. Indeed, Sirt1 activation could be downstream of AMPK, since AMPK activation has been related with an increase of intracellular NAD^+^ levels, which might promote Sirt1 activation [46], but it is not clear if the increase of NAD^+^ is AMPK-dependent in RSV treatments. Thus, it is difficult to establish if the probable interplay between AMPK and Sirt1 is reciprocal or one-way. However, growing evidence supports the hypothesis that activation of AMPK by RSV might occur independently of Sirt1 activity. This idea was strengthened by elegant experiments with AMPK recombinant insensitive to AMP protein, expressed in human embryonic kidney cells, where this AMPK recombinant protein was not activated by RSV treatment [6]. As mentioned, RSV increased the levels of AMP and ADP relative to ATP [6,35,36]. This data implies that the RSV-mediated inhibition of cellular respiration might cause an activation of AMPK by impairment in ATP production as a result of increased AMP/ADP levels. This mechanism coincides with the toxic effect attributed to RSV in plants. However, to support this idea further, evidence is required. Despite the fact that AMPK activation mechanism by RSV is still unclear, there is sufficient evidence to associate AMPK activation with the major physiological and improvements in CDDs caused by RSV treatment.

## 5. Amelioration of Chronic-Degenerative Diseases by Resveratrol

Several studies have demonstrated that nutritional supplementation of RSV in normal weight healthy subjects decreases the generation of reactive oxygen species (ROS), enhances the expression of both NAD(P)H:quinone oxidoreductase 1 (NQO-1) and glutathione *S*-transferase pi 1 (GST-P1) genes via nuclear factor-related factor 2 erythroid 2 (Nrf2), while it reduces the expression of intranuclear nuclear factor-κB (NF-κB) and suppresses plasma levels of inflammatory markers such as tumor necrosis factor-α (TNF-α), interleukine-6 (IL-6) and C-reactive protein (CRP) [47,48]. This is of major relevance since chronic inflammation is the hallmark of almost all CDD. The attenuation of this process has been associated with amelioration of CDD [49]. In this regard, it has been demonstrated that RSV reduces the development of non-alcoholic steatohepatitis (NASH), the hepatic manifestation of obesity and diabetes, in rats fed with a high-fat diet [50,51].

Other studies conducted on obese humans treated with 150 mg/day of RSV for 30 days, showed an improvement in serum lipid profile, blood glucose, triglycerides, alanine-aminotransferase, and inflammatory markers, mimicking the effects of DR [52]. Furthermore, cardioprotective effects of supplementation with a RSV-enriched grape extract (350 mg/day) for six months in 75 healthy patients showed a decreased in plasma levels of low-density lipoprotein (LDLc), apolipoprotein B (ApoB) and oxidized LDL (LDLox) [53,54]. Dyslipidemia is also a hallmark in several CDDs. Therefore, normalization of lipid levels has been associated with alleviation of CDDs by RSV supplementation.

Together, this data shows that administration of RSV alters the metabolic pattern related with the signaling pathway insulin receptor substrate 1/phosphatidylinositol 3-kinase/protein kinase B (IRS1/PI3K/AKT) mediated by AMPK. Insulin resistance, chronic inflammation, oxidative damage, and dyslipidemia are generally characterized by the activation of the IRS1/PI3K/AKT pathway, particularly, this being the route activated by insulin or insulin-like growth factor 1 (IGF-1) in insulin resistance [55]. This further activates the mechanistic target of rapamycin (mTOR), which subsequently activates p70-S6 kinase 1 (S6K1). S6K1 can phosphorylate and inactivate IRS1, thereby allowing a feedback regulation of the IRS1/PI3K/AKT pathway [56,57,58]. The absence of the S6K1 protein in mice protected them against obesity, enhanced β-oxidation, and improved insulin sensitivity, whereas two genetic models of obesity (mice *K/KA^y^* and *ob/ob*) fed with a high-fat diet showed markedly elevated S6K1 activity [58].

The IRS1/PI3K/AKT signaling pathway is considered a promoter of triglyceride accumulation in liver, due to its involvement on S6K1-mediated lipid metabolism. S6K1 can up-regulate by phosphorylation the transcription factor liver X receptor (LXR), a member of the nuclear receptors family [59]. Activation of LXR promotes transcription of the gene coding for sterol regulatory elements binding protein-1c (SREBP-1c), which is a transcription factor involved in the regulation of lipogenic enzymes such as acetyl-CoA carboxylase 1 and 2 (ACC) and fatty acid synthase (FAS) [59]. On the other hand, the inhibition of mTOR by rapamycin reduces the expression of lipogenic genes (*SREBP-1c/2*, *ACC*, *FAS* and esterearoil CoA desaturase 1 (*SCD1*)) [60,61,62]. Conversely, rat embryo cells with genotype *TSC1^−/−^* and *TSC2^−/−^* (corresponding to genes coding for TSC1/2 proteins) showed constitutive activation of mTOR and enhanced expression of lipogenic genes [62]. Together, this data indicates that hyperactivation of the IRS1/PI3K/AKT signaling pathway may promote mTOR activation, insulin resistance and hepatic triglyceride accumulation.

The deregulation of mTOR occurs in several human diseases, including cancer, obesity, diabetes type 2, and neurodegeneration [56]. Therefore, there are significant ongoing efforts to pharmacologically target this molecule [56]. The AMPK signaling pathway activated by RSV is antagonist to the IRS1/PI3K/AKT pathway. Although both pathways converge on mTOR, the first signaling pathway inhibits it, whereas the second actives it [56]. An interesting pharmacological approach for treating several CDD may rely on the AMPK pathway, since S6K1 remains inactive through inhibition by mTOR. Consequently, this may repress hepatic lipogenesis, insulin resistance, and inflammation. Additionally, this pathway leads to the inactivation of the transcriptional factor SREBP-2 responsible for the synthesis of cholesterol.

As mentioned before, increased levels of inflammatory cytokines are another important feature of several CDDs. The IRS1/PI3K/AKT pathway may also be related with such inflammatory status as AKT (also known as protein kinase B, PKB) phosphorylates and promotes the degradation of IKKα (lκB α kinase); this, in turn, allows for the translocation of NF-kB from the cytosol to the nucleus to promote the transcription of pro-inflammatory genes [63]. Thus, hyperactivation of AKT stimulates chronic inflammation, which could partially explain the high levels of inflammatory cytokines like TNF-α and IL-6 in numerous CDD [64].

**Figure 2:**
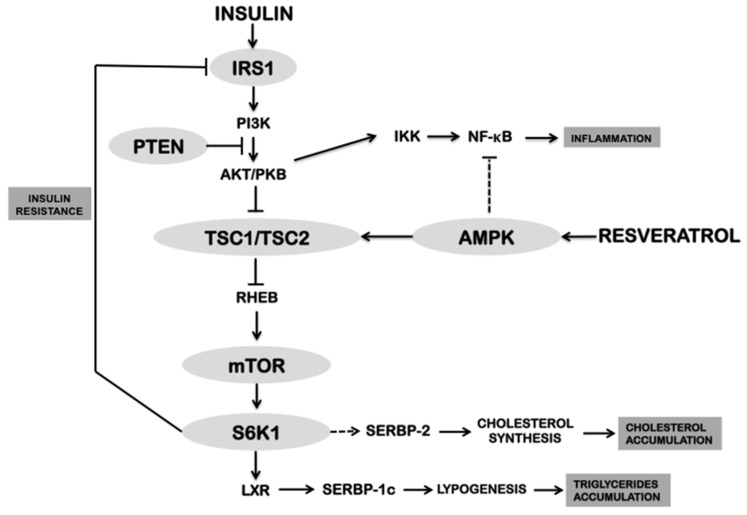
Integration of the physiological effects of resveratrol mediated by activation of AMPK and inhibition of IRS1/PI3K/AKT pathway. AMPK activation by resveratrol causes a pleiotropic effect and inhibits the anabolic pathway IRS1/PI3K/AKT, thus decreasing the accumulation of cholesterol and triglycerides, as well as improving the insulin resistance and inflammatory processes.

Overall, this data indicates that the health benefits of RSV supplementation depend on the inhibition of IRS1/PI3K/AKT pathway mediated by AMPK (Figure 2). However, supporting evidence for this hypothesis is still lacking and dose-response specific effects of RSV are even less understood.

## 6. Induction of Antioxidant Systems by Resveratrol

Regarding the biological responses by RSV, that can be divided into two groups, those exerted at low doses (<50 μM) and those at high doses (>50 μM). Higher doses of RSV promote mitochondrial dysfunction *in vivo* [65] and the pro-oxidant activity of this phytochemical. In contrast, low doses of RSV increase mitochondrial biogenesis [66], decrease ROS production in mitochondria and induce the overexpression of manganese superoxide dismutase (MnSOD) [9], thus acting as antioxidant molecule. It is possible that the contrasting effects of RSV could converge with the inhibition of cellular respiration and explain both distinct responses at molecular level.

**Figure 3:**
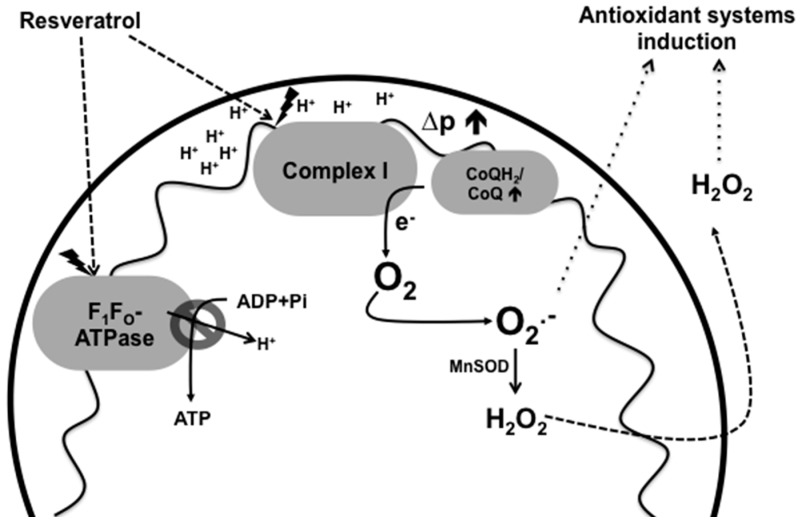
Pro-oxidant effects of resveratrol and its relation with the induction of antioxidant systems. The inhibition of complex I and/or F_1_F_0_-ATPase by RSV causes disengagement of the ETC with an increase in mitochondrial membrane potential (∆*p*) and production of O_2_^−^ and H_2_O_2_, as well as a decrease in ATP production and breathing. Increased ROS production could subsequently induce the antioxidant response as a defense mechanism.

It has been demonstrated that requirement or production of ATP diminishes electron transport in the ETC (decreased respiration). This, in turn, promotes a high protonmotive force in the mitochondria, increasing the NADH/NAD^+^ ratio and a favorably reduced coenzyme Q pool. These molecular changes promote O_2_^−^ generation by complex I [67] (Figure 3). Hence, it is expected that inhibition of the respiration by RSV stimulates the production of ROS within the cells. In this regard, it has been shown that RSV increases ROS production in *S. cerevisiae* [68] and in mammalian cell lines [69]. Furthermore, the *YAP1* mutant of *S. cerevisiae* (gen orthologous to mammalian AP-1 and the main antioxidant transcriptional factor in yeast) was more sensitive to RSV toxic effects in respect to the wild type. This indicates that RSV promotes cellular damage by ROS generation [68]. Therefore, the increase in ROS generation by RSV may be due to the inhibition of respiration, which subsequently might induce the antioxidant systems within the cells as a defense mechanism. To support this idea, it has been shown that RSV increases the catalytic activity of MnSOD in cardiac tissue of diabetic rats [70]. Similarly, RSV enhances the transcription of catalase and MnSOD genes in human mammary gland tumor cells [71].

Importantly, it has been reported that RSV behavior fits well with a hormetic response (defined as an relatively low exposition to oxidant challenge –“beneficial levels” of ROS- in the cells induces a long-standing antioxidant systems [72]) in several cell lines, where low-levels of RSV stimulate tumor cell proliferation whereas higher concentrations were inhibitory [73]. These experimental observations suggest that RSV at low doses allows the production of “beneficial levels” of ROS that stimulate cellular proliferation. In contrast, high doses of RSV promote a lethal increase of ROS causing mitochondrial dysfunction, which subsequently results in the release of cytochrome *c* and the induction of apoptosis.

## 7. Resveratrol and Mitochondrial Dysfunction

Mitochondria play a crucial role in metabolic cell functions. These complex organelles carry out a variety of processes including iron-sulfur cluster biogenesis, bioenergetics fluxes by Krebs cycle and ETC, apoptosis and regulation of antioxidant systems, among others [74]. As a consequence, mitochondrial dysfunction exerts pleiotropic effects in cells that may explain the tissue alterations seen in almost every pathological disease known [74,75].

Mitochondria have been considered the main source of ROS in most cells, with mitochondrial dysfunction leading to increased ROS generation, exhaustion of antioxidant defenses and manifestation of oxidative stress, being the latter phenomenon recognized as an important pathological mediator of several CDD [76,77]. Long-term oxidative stress leads to irreversible mitochondrial damage as might occur with high-doses of RSV. As mentioned before, mitochondrial dysfunction exerted by RSV induces the release of cytochrome *c* into the cytosol. This, in turn, activates the intrinsic mitochondria-mediated apoptotic pathway and by this mechanism the RSV inhibits tumor initiation and progression of a wide variety of malignant cells [78]. For example, lung cancer cell lines H838 and H520 treated with RSV (>50 µg/mL) exhibited a decrease of mitochondrial membrane potential, and liberation of cytochrome *c* followed by apoptotic death [79]. This suggests that high doses of RSV enhance the pro-oxidant properties of RSV, leading to mitochondrial dysfunction with drastic consequences for the cells. However, more evidence is necessary to support this concept.

## 8. Conclusions

The evidence discussed in this review allows us to propose that RSV inhibits cellular respiration, and this inhibition is the major effector of the molecular and physiological properties of RSV (Table 1). Although AMPK activation is crucial for the RSV-mediated beneficial effects in cells, it does not fully explain its toxic properties. We assume that the interplay of RSV with membranes probably can cause a negative effect on the catalytic region of ETC proteins and F_0_F_1_-ATPase. However, more conclusive evidence is needed to elucidate if the RSV-mediated inhibition of cellular respiration that we discuss in this review is really the convergent point of both beneficial and toxic properties. Therefore, it is necessary to comprehensively establish the mechanism of respiration inhibition by RSV.

Importantly, the information reviewed here indicates the toxicological potential of RSV supplementation. Therefore, more clinical trials targeted at specific diseases are needed to search for safe concentrations of RSV supplementation. For example, the toxicity exerted by high doses of RSV could help to treat cancer. On the other hand, risk factors of metabolic disorders related with energy overload as in high-fat and high-carbohydrate diets are ameliorated by low doses of RSV supplementation. This is probably due to activation of catabolic pathways mediated by AMPK. In this regard, subjects that consume high-energy diets accompanied by RSV supplementation could potentially have health benefits. Nonetheless, evidence about the effects of RSV supplementation under diets different than those with high-energy is still lacking.

## Figures and Tables

**Table 1:** Resveratrol targets sorted according to the mechanism proposed in this review.

Protein	Mechanism of Activation or Inhibition	Consequences	References
Complex I–III	Resveratrol and DUQH2 could act competitively on complex III	Inhibition of ETC, increase of ROS production	[30]
F_0_F_1_-ATPase	Inhibition of the rotatory mechanism of the F_1_-ATPase	Decrease in ATP production, increase of ROS production and activation of intrinsic mitochondria-mediated apoptotic pathway	[7,31,35,36]
AMPK	The increase of AMP-ADP levels due to inhibition of the ETC and F0F1-ATPase by resveratrol, activates the gamma subunit of AMPK	Activation of catabolism: stimulation of energy production from glucose and fatty acids. Inhibition of the IRS1/PI3K/AKT pathway	[6,11]
mTOR	Activation of AMPK by resveratrol inhibits mTOR through TSC1/2 activation	Inhibition of anabolism allows counteracting insulin resistance, cholesterol accumulation and dyslipidemia	[11,52,54]

## Abbreviations

The following abbreviations are used in this manuscript:

ACC: Acetyl-CoA carboxylase
ADP: Adenosine diphosphate
AKT: Protein kinase B
AMP: Adenosine monophosphate
AMPK: AMP protein kinase
ApoB: Apolipoprotein B
CDD: Chronic-degenerative diseases
CRP: C-reactive protein
DR: Dietary restriction
EPAC1: cAMP-regulated guanine nucleotide exchange factor
ETC: Electron transport chain
FAS: Fatty acid synthase
GST-P1: Glutathione S-transferase pi 1
HO-1: Heme oxygenase-1
IGF-1: Insulin-like growth factor 1
IKKα: lκB α kinase
IL-6: Interleukine-6
IRS1: Insulin receptor substrate 1
KEAP1: Kelch-like ECH-associated protein 1
NASH: Non-alcoholic esteatohepatitis
Nrf2: Nuclear factor-related factor 2 erythroid 2
LDLc: Low-density lipoprotein
LDLox: LDL-oxidized
LKB1: Liver kinase B1
LXR: Liver X receptor
mTOR: Mechanistic target of rapamycin
NF-κB: Intranuclear nuclear factor-κB binding
NQO-1: NAD(P)H quinone oxidoreductase 1
PDE4: cAMP phosphodiesterases
PGC1-α: Peroxisome proliferator-activated receptor γ coactivator
PI3K: Phosphatidylinositol 3-kinase
RSV: Resveratrol
RHEB: ras homolog enriched in brain
ROS: Reactive oxygen species
S6K1: p70-S6 kinase 1
SCD1: Esterearoil CoA desaturase 1
Sirt1: Sirtuin 1
SREBP-1c: Sterol regulatory element binding protein-1c
TNF-α: Tumor necrosis factor-α
TSC1/2: Tuberous sclerosis complex 1/2
